# Restriction and Sequence Alterations Affect DNA Uptake Sequence-Dependent Transformation in *Neisseria meningitidis*

**DOI:** 10.1371/journal.pone.0039742

**Published:** 2012-07-02

**Authors:** Ole Herman Ambur, Stephan A. Frye, Mariann Nilsen, Eirik Hovland, Tone Tønjum

**Affiliations:** 1 Centre for Molecular Biology and Neuroscience, Oslo, Norway; 2 Department of Microbiology, Oslo University Hospital (Rikshospitalet), Oslo, Norway; 3 Institute of Basic Medical Sciences, University of Oslo, Oslo, Norway; Saint Louis University, United States of America

## Abstract

Transformation is a complex process that involves several interactions from the binding and uptake of naked DNA to homologous recombination. Some actions affect transformation favourably whereas others act to limit it. Here, meticulous manipulation of a single type of transforming DNA allowed for quantifying the impact of three different mediators of meningococcal transformation: *Nla*IV restriction, homologous recombination and the DNA Uptake Sequence (DUS). In the wildtype, an inverse relationship between the transformation frequency and the number of *Nla*IV restriction sites in DNA was observed when the transforming DNA harboured a heterologous region for selection (*ermC*) but not when the transforming DNA was homologous with only a single nucleotide heterology. The influence of homologous sequence in transforming DNA was further studied using plasmids with a small interruption or larger deletions in the recombinogenic region and these alterations were found to impair transformation frequency. In contrast, a particularly potent positive driver of DNA uptake in *Neisseria sp.* are short DUS in the transforming DNA. However, the molecular mechanism(s) responsible for DUS specificity remains unknown. Increasing the number of DUS in the transforming DNA was here shown to exert a positive effect on transformation. Furthermore, an influence of variable placement of DUS relative to the homologous region in the donor DNA was documented for the first time. No effect of altering the orientation of DUS was observed. These observations suggest that DUS is important at an early stage in the recognition of DNA, but does not exclude the existence of more than one level of DUS specificity in the sequence of events that constitute transformation. New knowledge on the positive and negative drivers of transformation may in a larger perspective illuminate both the mechanisms and the evolutionary role(s) of one of the most conserved mechanisms in nature: homologous recombination.

## Introduction

Competence for transformation in the pathogenic *Neisseria meningitidis* and *Neisseria gonorrhoeae* has been studied for more than half a century and is a highly evolved and complex process where multiple proteins at the bacterial surface, in the membranes and in the cytoplasm are in contact with the transforming DNA [1]–[3], for reviews see [4]–[6]. Restriction modification systems (RMSs) impose a negative influence on the mobility of DNA in transformation by making double-strand breaks causing discontinuity and the disruption of homologous and potentially harmful heterologous sequences. RMSs are very common amongst bacteria and are constituted by a restriction endonuclease and a corresponding methylase which together provide a mechanism for limiting the import of unmethylated DNA. RMSs are abundant in the genus *Neisseria* [7]–[12]. It is debated if RMSs are selfish functional units or are under positive selection for their protective functions [13]. It has been argued that RMSs are surprisingly inefficient in representing only a modest barrier to inter-species recombination in both Gram-positive and Gram-negative species [14]. Restriction in *Bacillus subtilis* has been found to reduce transformation efficacy by only a factor of three, whereas the influence of sequence divergence between recipient chromosome and transforming DNA has been found to be a much stronger driver of sexual isolation [15]. Convincingly, transformation of both PCR products and chromosomal DNA harbouring *rpoB* alleles encoding rifampicin resistance enabled the definition of a log-linear relationship between sequence divergence and sexual isolation [15]. Recently, Budroni and co-workers demonstrated a remarkable correlation between the presence/absence of RMS and the phylogenetic structure of *N. meningitidis* [12]. Each phylogenetic clade (PC) of *N. meningitidis* was found to harbour a specific repertoire of RMS. Furthermore, since the stretch of DNA involved in gene conversion events was found to be longer in strains within PCs than between different PCs, an intriguing model was proposed in which RM is indeed an important player in the genesis and persistence of PCs. The impact of RMSs in the evolution of the highly recombinogenic meningococci makes a strong case for RMS as an efficient barrier towards DNA exchange and indeed a major driver of sexual isolation and speciation [12], at least in meningococci. Another factor to influence the fluidity of DNA by way of transformation in meningococci and gonococci is exerted by the DNA Uptake Sequence (DUS) [16], [17]. The DUS in *Neisseria* and the USS in *Pasteurellaceae* are generally considered a reproductive barrier that secures safe bacterial sex and species conservation by limiting DNA import to homologous alleles, contributing to sexual isolation [18]. It is well documented that incoming DNA containing a particular DUS is preferentially taken up during transformation [17], [19], [20] and that transformation is dependent on the type IV pilus machinery and RecA-mediated homologous recombination (HR) [21]–[23]. A positive correlation between the expression level of one of the minor pilins, ComP, and uptake of DUS-containing DNA has been described, but assembled pili or the pilin subunit itself do not display significant affinity for DNA [24], [25]. ComE, recombination proteins and other pilus-associated proteins such as the secretin PilQ have been found to be directly involved in transformation, however, the putative protein(s) directly responsible for DUS specificity still remains elusive [25]–[30]. The involvement of DUS in transformation is expected to be highly specific since abbreviated versions of DUS are dramatically impaired as mediators of transformation [29], [31], [32]. A linear relationship between the number of DUS and the ability to inhibit transformation in a competitive assay using a strain of *N. gonorrhoeae* has been demonstrated [33]. Furthermore, substantial variation between strains of *N. gonorrhoeae* with regard to DUS specificity and transformation efficacy has been documented, and it has been proposed that DUS may influence multiple steps during transformation [29] which may obscure the study of DUS activity and specificity.

The DUS sequence itself and the Uptake Signal Sequence (USS), which is found among the members of the family *Pasteurellaceae* [34], are both non-palindromic sequences, containing all four nucleotides, but they exhibit no apparent sequence identity to each other. The canonical decamer DUS (5′-GCCGTCTGAA-3′) is functional in transformation but the extended dodecamer (5′-AT-GCCGTCTGAA-3′, here named AT-DUS) elevates transformation further (17), a finding underpinning the notion that the putative protein-DUS interaction is expected to be highly specific.

The approximately 2000 copies of the DUS sequence itself make up nearly 1% of the meningococcal and gonococcal genomes and DUS is by far the most abundant decamer repeat present. Nearly 50% of the DUS in meningococcal genomes are arranged as inverted repeats and some constitute transcriptional terminators by forming stem-loop structures downstream of genes [17], [31]. The DUS stem-loop is not a required structure for the transformation process since a single DUS and a direct repeat DUS perform comparatively well in transformation [31]. However, it is unknown if the orientation or localisation of DUS may affect the transformation process. Since DUS are not palindromes, they are directional and when arranged as inverted repeats, one of the two possible arrangements is overrepresented: the stem-loop configuration DUS followed by reverse complementary DUS is far more common than reverse complementary DUS followed by DUS [35]. However, when DUS are located inside coding sequences (CDS), they appear to have a slight bias for the reverse complementary DUS [36]. This bias could possibly be explained by the presence of a stop codon in one reading frame of DUS not found in the reverse complementary DUS. Intragenic DUS also showed a biased distribution towards genome maintenance genes, and a genome-preserving function of DUS-mediated transformation was proposed as a driving force behind the evolution of both the DUS location bias and the extraordinary over-representation of DUS in the genome [36]. A comparative genomics study favoured the regenerative and conserving function of DUS, due to the frequent recombination and accumulation of DUS in permissive regions of the conserved core genome of three different neisserial species [37]. Another study analyzed 20 meningococcal genomes and revealed a significant correlation between the recombination rate and the density of DUS, confirming DUS as a marker of recombination [12]. DUS has also been shown to be closely associated to minimal mobile elements which utilise homologous recombination for chromosome integration [38]. Through evolution, DUS appears to have established itself due to the positive effect that transformation of homologous DNA exerts on genome stability by maintaining core functions [36], [37]. Thus, transformation may have evolved not only for generating adaptive variation, but also for its potential in recombinational DNA repair functions with a conservative output [39]. Reassembly of beneficial alleles in a single chromosome could be an efficient response to genotoxic stress and mutational load [40], [41]. Since most mutations are deleterious, the vast majority of beneficial alleles in transformation are expected to be non-mutated and well conserved alleles.

This study describes the negative influence exerted on transformation by *Nla*IV restriction and is quantified by manipulating the number of restriction sites that separates heterologous and homologous regions in the transforming DNA. In contrast, the influence of restriction was not observed during transformation of homologous DNA. The influence of the extent and integrity of homology in the transforming DNA was studied and shown to influence transformation substantially. Finally, the influence of DUS as a positive mediator of transformation was studied by delineating the effects of the quantity, orientation and location of DUS in transforming DNA. The additive effect of DUS was confirmed and a completely novel effect of DUS location documented. The characterization of these opposing drivers in meningococcal transformation provides an extended basis for understanding the dynamics of key steps in the process itself as well as the evolution of transformation and its barriers.

## Results

### *Nla*IV a type II Restriction Endonuclease in Meningococci

Out of 22 RMS identified in meningococci, the *Nla*IV RMS is one of the two most commonly occurring systems and is present in 19 out of 20 strains analyzed [12]. A BLAST search with the amino acid sequence of NMB1032, the *Nla*IV protein in N. *meningitidis* MC58, revealed that this gene is also common in *N. gonorrhoeae* strains and present in *N. lactamica*, *N. flavescens* and *N. polysacchareae* strains, but absent in all other sequenced species of the genus *Neisseria*. Other significant homologues (>50% identity) were found only in the genomes of *Treponema succinifaciens*, *Lachnospiraceae bacterium* and *Catenibacterium mitsuokai* indicating previous horizontal gene transfers across phylogenetically distant groups. *Nla*IV is a type II restriction endonuclease and a blunt-end cutter with the recognition sequence 5′-GGNNCC-3′. Based on the crystal structure of *Eco*RV, active site residues were defined in *Nla*IV and confirmed by *in vivo* and *in vitro* analysis of site-directed mutants [42]. The 2.2 Mb *N*. *meningitidis* MC58 genome has 1873 *Nla*IV sites and on average one *Nla*IV site per 1200 nucleotides. In MC58, the specific activities of three type II restriction endonuclease/methylase pairs in addition to *Nla*IV have been described [43]. These are *Nme*BI (*Hga*I homologue) with the recognition sequence 5′-GACGC-3′ [11], *Nme*B (*Eco*RII homologue) with the recognition sequence 5′-CCWGG-3′ [43] and *Nla*III (*Dpn*I homologue) with the recognition sequence 5′-GATC-3′ [44]. In addition, the MC58 genome harbours one Type I RMS (*Eco*RI24II), two type III RMS (*Eco*PI-ModB1 and *Eco*PI-ModA11) [12], [45] with uncharacterized specificities and several methylases without complimentary restriction endonucleases.

### Impact of *Nla*IV Restriction on Transformation

**Figure 1:**
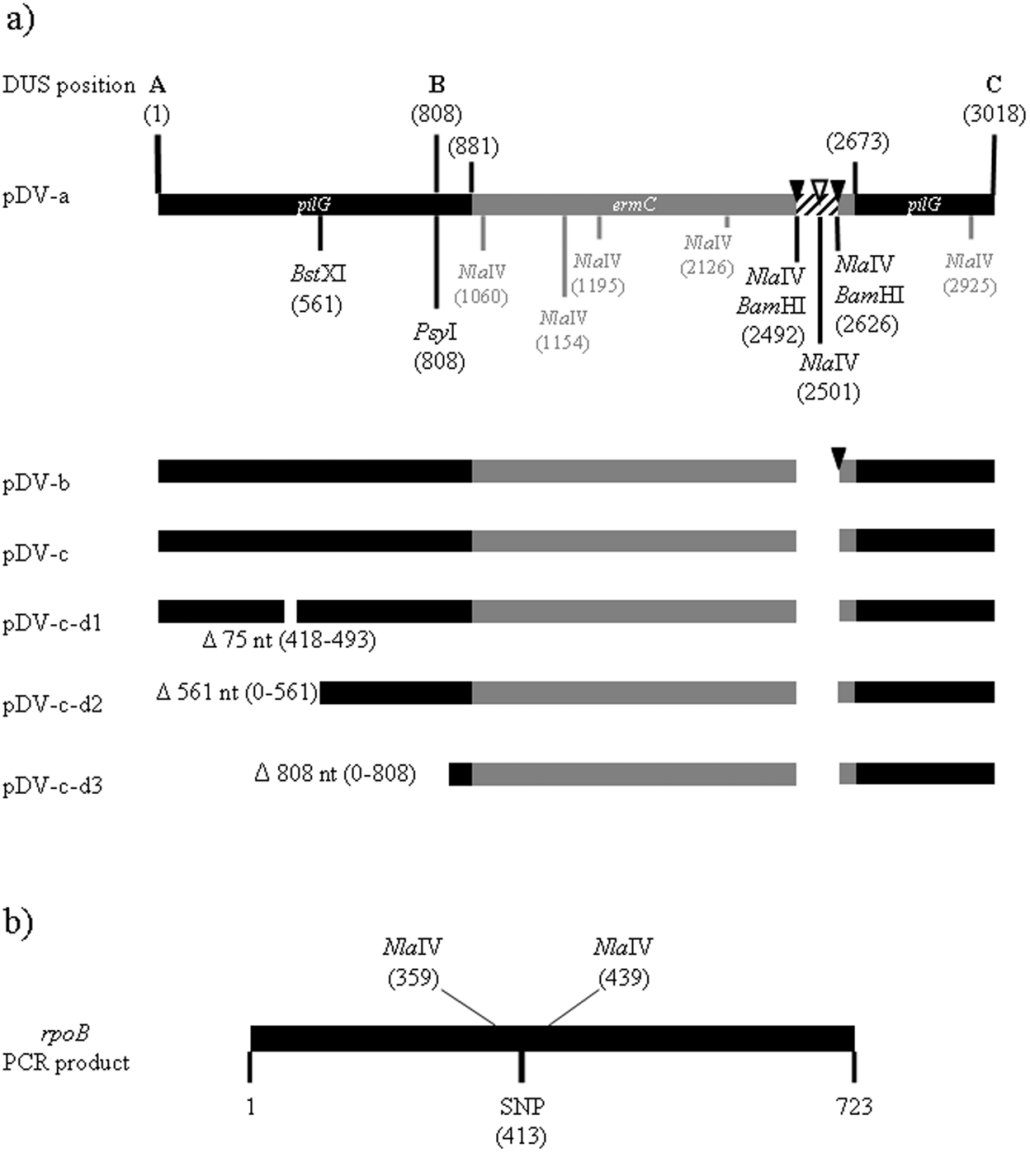
Sequence characteristics of transforming DNA. A. Variable DUS positions and restriction profiles in transforming DNA plasmids. Transforming DNA plasmids are based on *pilG* (black) interrupted by an erythromycin resistance insert, *ermC*, (gray). DUS (5′- ATGCCGTCTGAA-3′) or reverse complimentary DUS (5′-TTCAGACGGCAT-3′) are inserted in three different positions A, B and C as marked above the bar. All numbers refer to the nucleotide position following the start codon (1) of *pilG.* The 137 nucleotide long *Bam*HI-fragment which is removed in pDV-b, pDV-c versions is shown in white with black stripes. **B.** ***Nla*** **IV restriction profile of** ***rpoB*** **and location of selective SNP.** The homologous 723 nt long PCR fragment of an internal part of the meningococcal *rpoB* gene used in transformation contains two *Nla*IV restriction sites on both sides of the selective SNP responsible for rifampicin resistance in the recipient.

**Figure 2:**
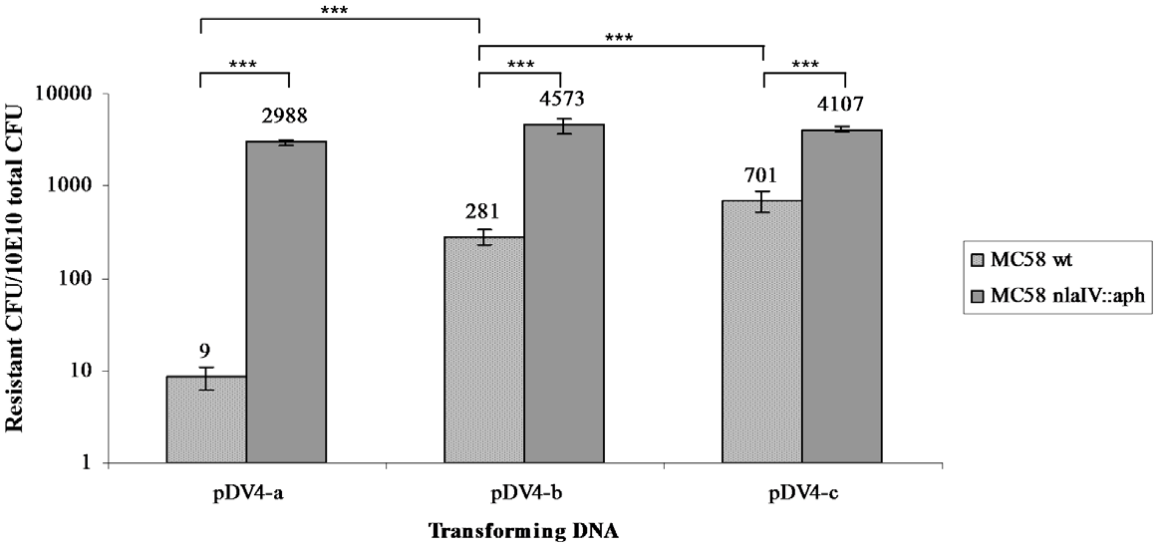
*Nla*IV restriction affects plasmid transformation in *N. meningitidis* MC58 wildtype and not the *Nla*IV null mutant. The Y axis shows the number of resistant (erythromycin) CFU/total 10^10^ CFU on a log scale. Along the X-axis are the different DNA substrates (10 ng/ml) with altered numbers of *Nla*IV restriction sites shown. pDV4-a harbours two and three *Nla*IV restriction sites more than pDV4-b and pDV4-c, respectively. For further details of the restriction profiles and DUS locations in the transforming DNA plasmids please consult Figure 1 and Table 2. Statistically significant differences in transformation frequencies between the *Nla*IV null mutant and wildtype backgrounds are indicated above the columns, ***equals p≤0.001 in a two tailed paired student’s t-test.

**Figure 3:**
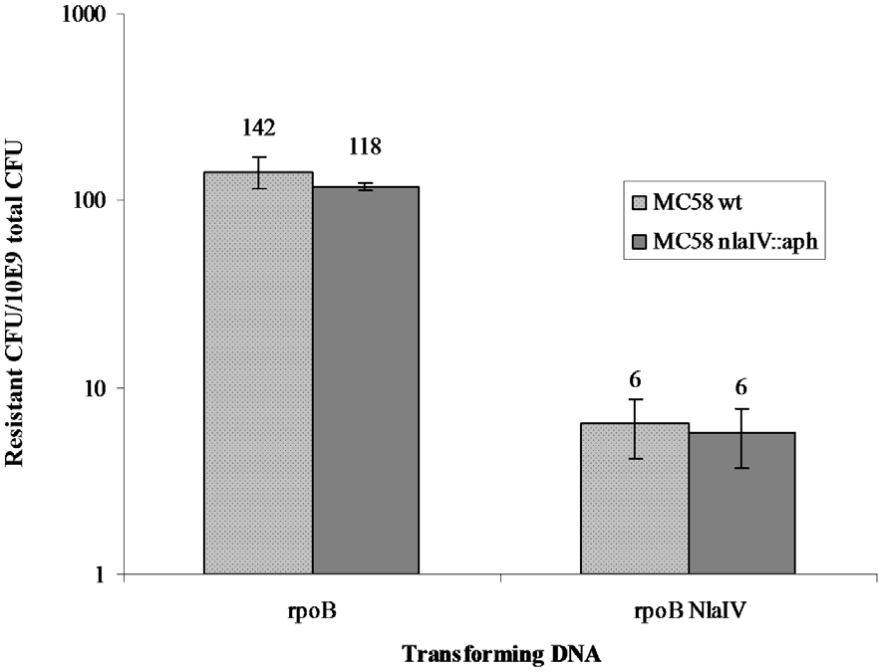
*Nla*IV restriction does not affect homologous DNA transformation in *N. meningitidis* MC58 wildtype or in the *Nla*IV null mutant. The Y axis shows the number of rifampicin-resistant CFU/total CFU 10^9^ on a log scale. Along the X-axis are shown the two different DNA substrates (5 ng/ml), *rpoB* PCR fragment and *rpoB* PCR fragment pre-digested with *Nla*IV. For further details on the transforming DNA please consult Figure 1 and Table 2. The transformation frequencies in the wildtype and in the *Nla*IV null mutant backgrounds are not statistically significant from each other.

Three different versions (-a, -b and -c) of plasmid pDV4 (w/DUS in position A) with reducing numbers of *Nla*IV restriction sites as shown in Figure 1, were tested for their ability to transform wildtype *N. meningitidis* MC58 and its *Nla*IV null mutant. The pDV4-a plasmid has eight *Nla*IV sites in the *pilG*Δ*ermC* region, three of which lie in the region which is connecting the 3′ terminal part of *pilG* and the selective marker (*ermC*) as shown in Figure 1. The pDV4-b plasmid has a single *Nla*IV site in the connecting area where the pDV4-c version has none, as shown in Figure 1. The transforming abilities of individual plasmids on meningococcal wildtype and the *Nla*IV null mutant were tested in a liquid culture transformation assay and the results are shown in Figure 2. Compared to the pDV4-a, a more than 30-fold higher transformation frequency was observed when using pDV4-b (p≤0.001, two-tailed paired student’s t-test). A 70-fold increase was observed when using pDV4-c compared to the frequency obtained with pDV4-a. The pDV4-b and pDV4-c versions differ only in the presence/absence of a single *Nla*IV restriction site respectively, and this difference had >2.5-fold effect on the transformation frequency (p≤0.005, two tailed paired student’s t-test). Furthermore, all plasmids transformed at considerably higher frequencies in the *NlaIV* background. This was particularly pronounced with transformations using pDV4-a which performed most poorly in the wildtype and more than hundred-fold better in the *Nla*IV null mutant. The pDV4-b plasmid transformed sixteen-fold better in the *Nla*IV null mutant as compared to the wildtype and the pDV4-c plasmid more than five-fold better. The >2.5-fold difference between -b and -c plasmids documented in the MC58 wildtype strain was not seen in the *Nla*IV null mutant background. The different performance of pDV4-a and pDV4-b in the *Nla*IV mutant background are not statistically significant. Since the plasmids employed in this study contain large heterologous regions harbouring most of the *Nla*IV restriction sites, we were encouraged to investigate if the negative influence on transformation by *Nla*IV restriction also could be detected when using homologous DNA. Several genes conferring different types of resistance in Mc by way of a single nucleotide change were considered, however, only in *rpoB* did we find the selective mutation to be “tightly locked in” by surrounding *Nla*IV sites as shown in Figure 1 B. A 723 nt internal fragment of *rpoB* encoding rifampicin resistance was therefore amplified by PCR using primers containing DUS and used in liquid culture transformation of both wildtype and *Nla*IV null mutant strains. The results are shown in Figure 3. In contrast to the pDV-transformations, both the wildtype and the *Nla*IV null mutant were transformed equally well by the homologous *rpoB* fragment. The *Nla*IV-digested *rpoB* fragment served as a control and had a twenty-fold reduced ability to facilitate transformation compared to the intact *rpoB* fragment.

### Influence on Transformation of the Integrity and Size of the Recombinogenic Region in Transforming DNA

**Figure 4:**
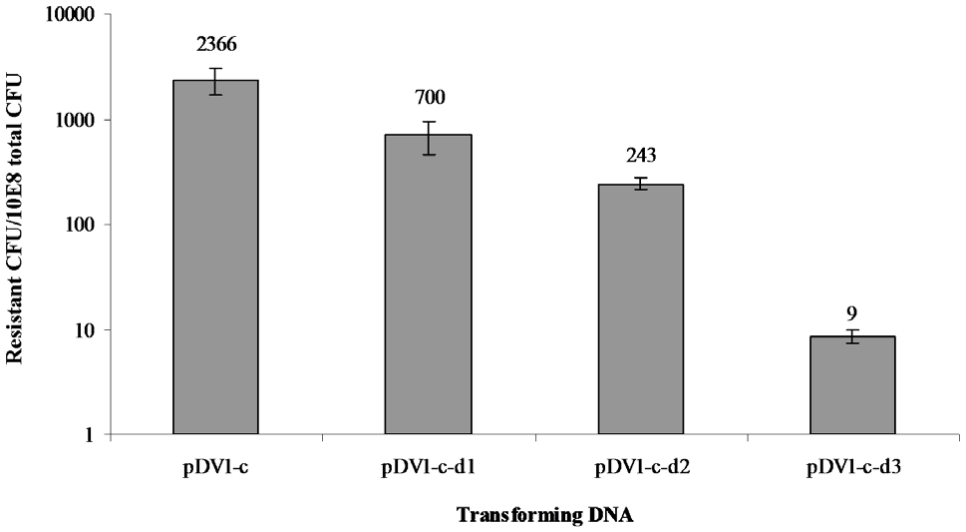
Deletions in the recombinogenic region of plasmids impose a negative influence on transformation. The Y axis shows the number of erythromycin-resistant CFU/total CFU 10^8^ on a log scale. Along the X-axis are shown the different transforming plasmids (1 µg/ml) with altered regions of homology. For further details on the transforming DNA please consult Figure 1 and Table 2. Differences in transformation frequencies are statistically significant from each other as indicated above the columns, **equals p≤0.05 in student’s t-tests.

Since it appeared that the pDV plasmids and the *rpoB* fragment were influenced differentially by *Nla*IV restriction during transformation, we became interested in the influence of homology on transformation. Furthermore, since several processes influence transformation and a whole range of different plasmids traditionally has been used to monitor transformation frequencies in meningococci, obtaining insight into the relative influence of distinct homology alterations seemed important. Three new pDVs were therefore made by deleting parts of the homologous region upstream of the *ermC* insert as shown in Figure 1. In pDV1-c-d1, a 75 nt deletion was introduced at an approximate middle of the upstream region, in pDV1-c-d2, the first 561 nt were completely removed and in pDV1-c-d3, most of the upstream region was deleted leaving only 73 homologous nt to facilitate HR. The distance from the internal deletion to the heterologous *ermC* insert is 395 nt in pDV1-c-d1, while and in pDV1-c-d2, 320 nt remains. As shown in Figure 4 these deletion plasmids transform the *Nla*IV null mutant progressively at lower frequencies than the intact pDV1-c. Compared to pDV1-c, the pDV1-c -d1 plasmid with the 73 nt internal deletion transform at only 34%. The pDV1-c–d2 and pDV1-c–d3 with larger deletions transformed with frequencies one and two orders of magnitude less than pDV1-c, respectively.

### The Effects of DUS Directionality, Location and Multiplicity on Transformation

**Figure 5:**
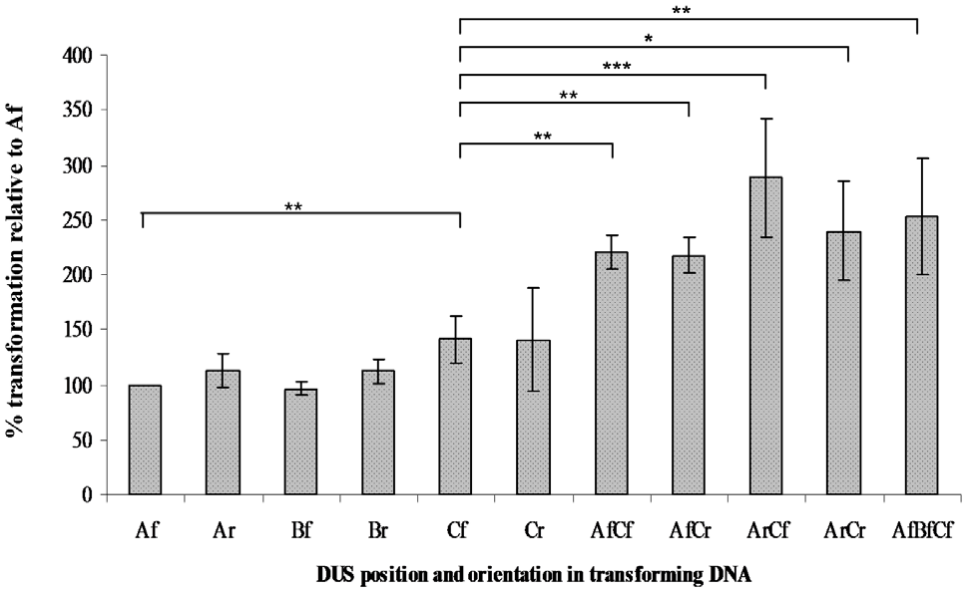
Effects of DUS orientation, location and multiplication on transformation. The Y axis shows the efficacy of transformation as percent of the transformation obtained with the internal standard Af plasmid (pDV4-c). Along the X-axis are shown the different DNA substrates (10 ng/ml) identical in all but DUS in three different positions (A, B and C), in two different orientations, forward (f) and reverse (r), and in the combinations of these. For further details on the DNA plasmid templates please consult Figure 1 and Table 2. Statistically significant values are indicated above the columns, **equals p≤0.05 and ***equals p≤0.001 in a two-tailed paired student’s t tests.

Once the effect of *Nla*IV restriction on transformation of the pDVs containing a heterologous region was established, we were curious to see if also a possible phenotype regarding the orientation, location and number of DUS could be found by using similar plasmids. A recent report demonstrated that DUS specific binding and uptake not fully correlated with DUS dependent transformation, suggesting that DUS influenced more than one level of the transformation process [29]. One such level following binding and uptake may be DUS-initiated DNA processing in some form. If DUS were to initiate processing of the test plasmids in a linear fashion during transformation, the influence of *Nla*IV restriction on transformation could potentially differ between plasmids harbouring DUS in the three different positions (Figure 1), since *Nla*IV recognition sites are asymmetrically distributed. No previous study has investigated if DUS orientation relative to homologous regions of DNA may influence the fate of transforming DNA. Furthermore, a previous study showed that DNA with DUS spaced very closely together (5 nt apart) did not significantly transform neisserial (gonococci and meningococci) cells at higher efficiency than an identical construct with a single DUS [31]. A remaining question was if transformation efficacy could be affected if multiple DUS were more dispersed and if the variable placement and orientation of DUS were influencing factors. In the meningococcal chromosome, DUS are on average spaced 1 kb apart [46]. However, due to the high percentage of closely spaced DUS occurring as inverted repeats, a more true average distance between DUS locations is 1.5 kb. A set of pDVs (all-c) was therefore employed with DUS inserted into one of the two possible orientations (forward and reverse) in three dispersed positions (A, B and C) and in combinations of these, to test their performance in transformation of *N. meningitidis* MC58 as above [31]. The DUS positions were: position A at the 5′ end of the homologous region and selective marker, position B also 5′ to the selective marker but inside the homologous region (*pilG*) and position C at the 3′ end of the homologous region and selective marker (Figure 1A). First, as shown in Figure 5, no effect of altering the orientation of DUS could be observed by comparing the performances of plasmids harbouring DUS in one of the two possible orientations (forward 5′-AT-GCCGTCTGAA-3′ and reverse 5′-TTCAGACGGC-AT-3′) but were otherwise identical. The same insensitivity to DUS orientation was observed for all three different DUS locations. Secondly, a weak tendency for the plasmids with DUS in position C to outperform the plasmids with DUS in position A or B was observed (Af to Cf comparison, p≤0.005, paired two tailed student’s t-test). Finally, a clear increase in transformation frequency was observed when the numbers of DUS were doubled. All frequencies obtained with plasmids containing multiples of DUS were significantly different from the single Af plasmid (pDV4-c).Also, by comparing the performances of plasmids with multiples of DUS to that of the Cf plasmids (pDV1-c), the differences are significant (p≤0.005, paired two tailed student’s t-test, p = 0.0575 in Cf to ArCr comparison) as shown in Figure 5. The additive effect of DUS in the plasmids assayed here seemed to plateau since the construct with three DUS does not transform threefold better than the single DUS construct but rather displayed the same frequency as the constructs with two DUS.

**Figure 6:**
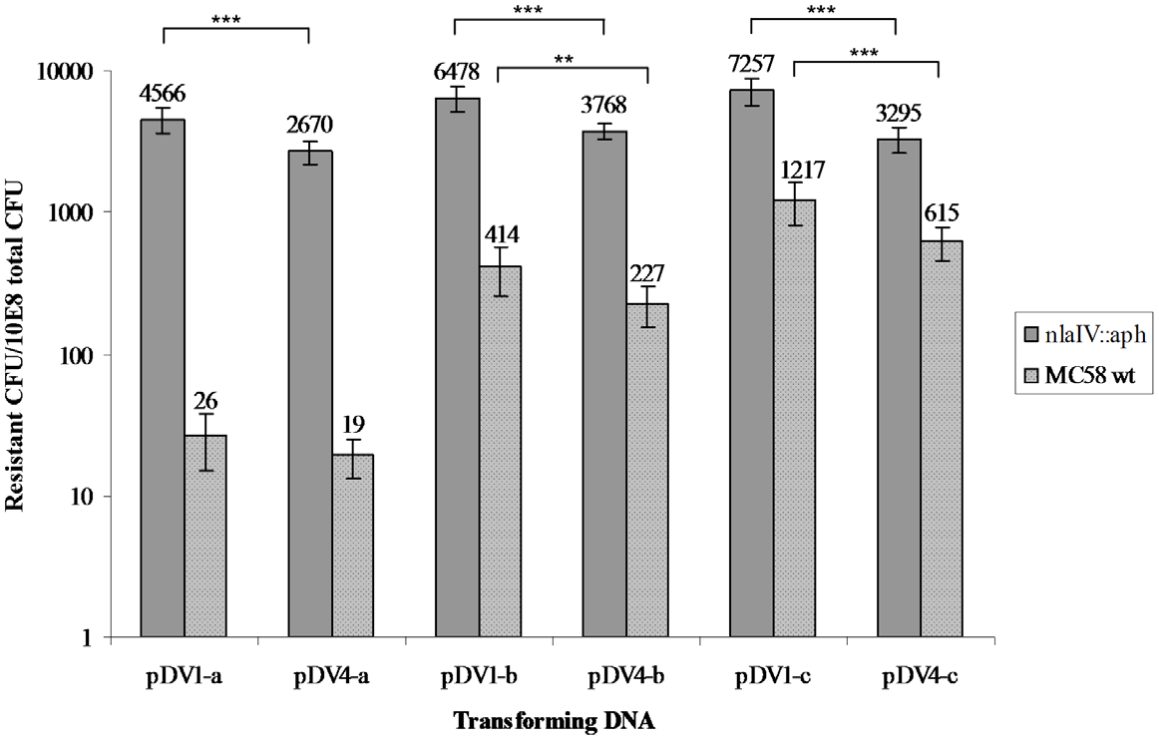
The influence of DUS location on transformation. The Y axis shows the number of erythromycin-resistant CFU/total CFU 10^8^ on a log scale. Along the X-axis are shown the transforming DNA (1 µg/ml) that differ in *Nla*IV restriction profiles and DUS location. For further details on the DNA plasmid templates please consult Figure 1 and Table 2. Statistically significant values are indicated above the columns, **equals p≤0.05 and ***equals p≤0.001 in a two-tailed paired student’s t tests.

The effect of varying the location of DUS relative to the recombinogenic region was unexpected and warranted closer investigation. A number of transformations were therefore performed using all versions of plasmids with DUS in position A (pDV4-a, pDV4-b and pDV4-c) or in position C (pDV1-a, pDV1-b and pDV1-c) in both wildtype and *Nla*IV mutant backgrounds. As shown in Figure 6, plasmids with a DUS in position C outperformed the plasmids with a DUS in position A in both backgrounds and in all but one pairwise comparisons. In the *Nla*IV mutant background pDV1-a with DUS in position C transformed 1.7-fold higher than pDV4-a with DUS in position A. Similarly, pDV1-b transformed 1.7 times higher than pDV4-b and pDV1-c transformed 2.2-fold higher than pDV4-c with statistical significance in the *Nla*IV mutant background as shown in Table 1. Also in the wildtype background, a slightly less than two-fold effect on transformation was observed in the pDV1-b to pDV4-b and pDV1-c to pDV4-c comparisons. The only pairwise comparison of DUS in position C with DUS in position A with an insignificant difference was the 1.3-fold higher performance of pDV1-a relative to pDV4-a in the wt background, as shown in Table 1.

**Table 1:** Fold-differences and statistical significance values (paired two tailed student’s t tests) in comparing the performances of individual plasmids harbouring DUS in the C (pDV1) or A (pDV4) positions in transformation of the *Nla*IV mutant and wt backgrounds.

MC58 ΔnlaIVR			
	pDV1-a	pDV1-b	pDV1-c
pDV4-a	1.7 fold, p = 0.0002 (N = 8)	–	–
pDV4-b	–	1.7-fold, p≤0.0001 (N = 8)	–
pDV4-c	–	–	2.2-fold, p≤0.0001 (N = 8)
MC58 wt			
pDV4-a	1.3-fold, p = 0.1203 (N = 10)	–	–
pDV4-b	–	1.8-fold, p = 0.0044 (N = 10)	–
pDV4-c	–	–	1.9-fold, p = 0.0004 (N = 10)

N denotes the number of replicate experiments.

## Discussion

For successful transformation of DNA into the meningococcal cell, a set of well-defined conditions must be in place. Firstly, transformation requires a multi-component competence machinery in the recipient (reviewed in [4]). Secondly, DUS is generally required for efficient transformation, although DUS-independent transformation has been documented in certain gonococcal strains [29], [32]. Previous work has shown that transformation in *N. meningitidis* MC58, the strain used in this study, is strictly DUS-dependent, since DUS-less DNA fails to transform this strain at any significance. In the case when a heterologous region of DNA is transformed the length and similarity of flanking homologous regions are important for achieving chromosomal integration.

It has been shown in *Neisseria sp.* that plasmid DNA is restricted during transformation, but not in conjugation [47], [48]. In a co-cultivation experiment, Claus and co-workers showed that restriction by the restriction endonuclease *Nme*BI caused a near four-fold decrease in transformation from one meningococcal strain to another [11]. The data presented here suggest that *Nla*IV restricts transforming plasmid DNA and that this restriction has a negative effect on the transformation frequency. A ≥2.5-fold effect on transformation by removing a single *Nla*IV restriction site and more than thirty-fold effect of deleting a 137 nt long heterologous DNA segment containing three *Nla*IV sites in the transforming DNA, were demonstrated. In addition to the three *Nla*IV sites that were monitored for their effect on transformation, there are yet five additional *Nla*IV sites in the pDV4 plasmids tested. Their influence was emphasized by the considerably higher transformation frequencies of all three plasmids in the *Nla*IV mutant background. The five-fold difference between the transformation frequencies of the pDV4-c in the wildtype and null mutant can be explained by the lack of restriction in the null mutant in the four remaining *Nla*IV sites inside the selective marker, as depicted in Figure 1. These observations suggest that the effect of *Nla*IV restriction on the tested transforming DNA is highly cumulative. The explanation for the negative effect on transformation is likely the dissociation from homologous regions and the disintegration of the *ermC* resistance marker, which is required for selection. This is further supported by the observations that the homologous *rpoB* fragment was not influenced to a detectable extent by *Nla*IV restriction. A possible explanation for the discrepancy observed between transformations with homologous and partly heterologous DNA is that reduced efficacy of HR allows prolonged or more efficient restriction of transforming DNA. In the current models for bacterial transformation, DNA is recovered in a double-stranded (ds) form inside the cell [49], but enters the cytoplasm as single-stranded (ss) DNA, as in conjugation [50]. Since restriction endonucleases generally only act on dsDNA, it remains unclear how RMS in the cytoplasm can affect transforming DNA and highlights a gap in our understanding of transformation [47]. Elaborating on the strategies employed here may be helpful in targeting the exact events that take place upon DNA entry into the cytoplasm. The formation of ssDNA has been detected during transformation in *N. gonorrhoeae* and explanatory models where restriction sensitive dsDNA is formed in recombination intermediates or in self-complimentary regions on ssDNA have been proposed [51], [52]. Restriction of ssDNA by Type II restriction endonucleases has been documented although at a lower rate than for dsDNA [53]. Restriction endonucleases might also act on dsDNA after alignment of the incoming DNA with homologous regions of the genome in the recombination process when DNA is still only hemi-methylated. We do not particularly favour the hypothesis that reduced transformation of the pDVs employed here are caused by restriction of ssDNA since it is difficult to envisage that RMSs have evolved such a high specificity for dsDNA compared to ssDNA, if the evolutionary benefit of RMSs is to restrict transforming DNA. On that note it is very interesting that we were unable to detect restriction of a homologous DNA fragment. This observation could indicate that the evolutionary benefits of RMSs are linked to their abilities in limiting the import of heterologous and potentially harmful DNA and are less important for limiting transformation of homologous DNA. However, as detailed above, Budroni and co-workers found a correlation between the phylogeny of meningococcal lineages and their respective RMS repertoires indicating abundant restriction of homologous DNA during transformation. It is therefore possible that experimental set-ups, such as the liquid culture transformation assay used here, are not fully able to detect more subtle events that shape meningococcal genomes in an evolutionary time-frame. Also, the added influence of multiple RMS could be expected to drive the progressive divergence of lineages. Further analyses addressing particularly the ssDNA/dsDNA conundrum are required to deconstruct the specificities of restriction events in *Neisseria* sp. and other competent bacterial species.

**Table 2:** Plasmids and bacterial strains.

Plasmids	Relevant characteristics, DUS position(s) and orientation(s) in ()	Source
pBluescript II SK+	General cloning vector, amp^r^, abbreviated pBSK+	Stratagene
p0-DUS-a	pBSK+ harbouring *pilG*::mTnErm	[31]
p0-DUS-b	p0-DUS with 137 nt removed incl. two *Nla*IV sites removed	This study
p0-DUS-c	p0-DUS-b w/one *Nla*IV site removed	This study
pSingle	pBSK+ harbouring *pilG*::m*Tn*Erm w/forward DUS is *Psy*I site at pos. 808 (Bf)	[31]
pOHA13-c	pSingle w/137 nt and and three *Nla*IV recognition sites removed (Bf)	This study
pOHA13r-c	pOHA13-c with DUS reversed (Br)	This study
pDV1-a	p0-DUS-a w/DUS in position C (Cf)	
pDV1-b	p0-DUS-b w/DUS in position C (Cf)	
pDV1-c	p0-DUS-c w/DUS in position C (Cf)	This study
pDV-c-d1	pOHAD1-c w/74 nt deletion from 396 nt into *pilG* gene from start	This study
pDV1-c-d2	pDV1-c w/561 nt deletion from *pilG* gene start	This study
pDV1-c-d3	pDV1-c w/808 nt deletion from *pilG* gene start	This study
pDV4-a	p0-DUS-a w/forward DUS in position A (Af)	This study
pDV4-b	pDV4-a w/137 nt removed incl. two *Nla*IV sites	This study
pDV4-c	pDV4-b w/one *Nla*IV site removed	This study
pDV5-c	p0-DUS-c w/forward DUS in positions A and C (AfCf)	This study
pDV6-c	p0-DUS-c w/forward DUS in positions A, B and C (AfBfCf)	This study
pDV54	p0-DUS-c w/reverse DUS in positions A (Ar)	This study
pDV55	p0-DUS-c w/reverse DUS in positions C (Cr)	This study
pDV57	p0-DUS-c w/reverse DUS in positions A and C (ArCr)	This study
pDV58	p0-DUS-c w/reverse DUS in positions A and forward DUS in position C (ArCf)	This study
pDV59	p0-DUS-c w/forward DUS in positions A and reverse DUS in position C (AfCr)	This study
pOHA1032::*aph*	pBSK+ with partial *nlaIVR* (NMB1032) interrupted by kanamycin resistance gene *aph* from pUP6	This study
pUP6	Substrate for PCR of kanamycin resistance gene *aph*	[62]
**Strains**		
*Escherichia coli* XL-1 Blue	*endA1 gyrA96(nal^R^) thi-1 recA1 relA1 lac glnV44 F’[::Tn10 proAB^+^ lacI^q^ Δ(lacZ)M15] hsdR17(r_K_^−^ m_K_^+^)*	Stratagene
*Neisseria meningitidis MC58*	*Serogroup B isolated in England*	[63]
*Neisseria meningitidis* MC58Δ*nlaIVR*	Restriction endonuclease *Nla*IV (NMB1032) null mutant of *Neisseria meningitidis* MC58	This study

**Table 3:** Primers.

Primer name	Primer sequence	Usage	Reference
3892OH11_*pilG*5′*Xho*I	ACGACTCGAGATGGCTAAAAACGGAGGAT	Multiple plasmids	[31]
8907OHA11_DUS	ACGACTCGAGATGCCGTCTGAAATGGCTAAAAACGGAGGAT	w/DUS in pos. Af	This study
3893OH3	TAGACCGCGGTCAGGCGACCACGTTGCC	Multiple plasmids	
8908OHA3_DUS	TAGACCGCGGTTTCAGACGGCATCAGGCGACCACGTTGCC	w/DUS in pos. Cf	This study
11368SF129	ACGACTCGAGTTTCAGACGGCATTGGCTAAAAACGGAGGAT	w/DUS in pos. Ar	This study
11369SF130	TAGACCGCGGATGCCGTCTGAAACAGGCGACCACGTTGCC	w/DUS in pos. Cr	This study
10324OHA2020	GCCTCGAGAATACGATTTATTGGGCAATACCGTTG	pOHA1032::*aph*	This study
10325OHA2021	AGGCTAGCTGAATGATGTTGCCGACGACATC	pOHA1032::*aph*	This study
10326OHA2022	GCGAATTCTACGATATGGACGACAACGGCAAT	pOHA1032::*aph*	This study
10327OHA2023	GCTCTAGATCAATTGCGGAAACAAAATCTTCCAA	pOHA1032::*aph*	This study
10327OHA2023	GCGAATTCTCATTTCGAACCCCAGAGTC	pOHA1032::*aph*	This study
8184OHA_*AphEco*RI_REV	GCGAATTCTCATTTCGAACCCC-AGAGTC	pOHA1032::*aph*	This study
12950OHA2187	TTCGGAGACATTTCAGACGGCATTTGTCCGCAAAGGGACGATT	w/DUS in pos. Br	This study
12951OHA2188	GCTTGCGGACAAATGCCGTCTGAAATGTCTCCGAAAATCGGCA	w/DUS in pos. Br	This study
S1	TTTTGGTCGTAGAGCACACG	Sequencing	[31]
S2	AAACATGCAGGAATTGACGA	Sequencing	[31]
S3	TCGGTTTGGTATTCGTGATG	Sequencing	[31]
PR2488	GTAAAACGACGGCCAGT	Sequencing	[31]
PR2487	AGCGGATAACAATTTCACACAGGA	Sequencing	[31]
13573OHA2220	TACAGTCCATGGTTGCAGTAGAAGCGGTCGAA	Version –d	This study
13574OHA2221	AACCCATGGCCATCCGCAAAAAGGT	Version –d	This study
31567OHA2217	TTCAGACGGCATATACGCGCACATACGAACAA	*rpoB* fragment	This study
31568OHA2218	ATGCCGTCTGAACAATCACATAGCGGCCTTCT	*rpoB* fragment	This study

It was evident from the restriction analysis that homologous DNA interrupted by heterology was less efficient in transformation than a continuous stretch of homologous DNA. To which extent the amount of homologous sequence itself influenced the efficacy of transformation was therefore studied by using three versions of a test-plasmid in which the homologous sequence was altered. Previous studies have described that regions ranging from 25–200 nt of sequence with high similarity to the recipient genome are required for HR [18]. However, in this study there were more than 300 nt remaining of continuous perfect homology between the pDV1-c–d1 plasmid and the recipient genome and yet the 74 nt internal deletion had a > three-fold negative influence on transformation. HR therefore seems prone to terminate upon encountering homologous sequence discontinuity, even when the discontinuity is caused by a small internal deletion. Some continuation of HR seems to be allowed past the deletion since the pDV1-c–d1 plasmid performed near three times better than the pDV1-c–d2 plasmid with a comparable 561 nt complete truncation up to the same region. The distance from *ermC* to the internal deletion in pDV1-c–d1 (388 nt) is 68 nt longer than the equivalent distance in pDV1-c–d2 (320 nt), and is unlikely to account for the near threefold difference in transformation performance observed with pDV1-c–d1 and pDV1-c-d2, assuming the absence of particular biases for cross-overs in the short stretch. The plasmid pDV1-c–d3 with the largest truncation (808 nt) allowed for very little transformation, emphasizing that heterologous sequences require flanking homologous sequence to be integrated into the chromosome. It seems that the remaining 75 nt of homologous sequence in pDV1-c–d3 is approaching the limits for efficient HR as defined by previous studies [18]. This means that, within the limitations of the assay, transformation of the pDV plasmids likely requires two cross-over events, one on either side of the selection marker. These observations may be helpful in the design of DNA for chromosome manipulations by way of transformation in meningococci and other competent species.

Finally, since asymmetry in both restriction sites and sequence homology was shown to influence transformation, we were enticed to investigate if asymmetric distribution of DUS also could influence transformation. First, it was demonstrated that both orientations (forward and reverse) of DUS performed equally well in transformation. These results suggested that DUS is not a starting point for directional DNA processing during transformation. Rather, a DUS-initiated process could possibly relate to the DNA molecule in either a random or bi-directional manner. It is possible, however, that if DUS influences several steps during transformation [29], some DUS-specific interactions are exerting stronger influence on the transformation process than others, masking a potential directional bias. The transformation frequencies obtained with the plasmids harbouring two (and three) DUS were found to double, in line with previous studies [33]. A pioneering study using a competitive assay has suggested a linear correlation between the number of DUS and the ability to inhibit transformation [33]. The plasmids employed in many previous transformation studies [17], [33] differed in characteristics to the extent that they, with our current understanding of factors that can influence transformation from the data presented here, were not directly comparable. These characteristics are the length of DUS (DUS vs. AT-DUS), RMS profile, DUS location, lengths of homologous regions and size of donor DNA fragments. Furthermore, since the results of the DNA binding and uptake assay have recently been shown to not fully correlate with the outcome of transformation assays [29], the transformation set-up presented here may therefore better represents the totality of events in the transformation process since it also considers the final step, HR. [54], [55]. One could perhaps suspect that, if DUS specificity was solely surface located, e.g. on a pilin subunit such as ComP, and access to homologous DNA was a limiting factor, increasing the number of DUS would improve the likelihood for a piece of DNA to make initial sequence-specific contact and consequently be guided into the transformation pathway. Extracellular DNA may not be in short supply since gonococci, meningococci and other *Neisseria sp.* reside in biofilms [56] which could be rich in DNA due to cell lysis and DNA secretion [57]–[59]. Surprisingly, the study of the panel of single DUS constructs also documented that the location of DUS relative to the two recombinogenic regions and the selective marker did influence the efficacy of transformation. A tendency for the plasmids with DUS in position C to outperform those with DUS in position A (1.8-fold) was observed, both in wildtype and in the *Nla*IV null mutant background. This tendency was observed for all three pairwise comparisons of plasmids differing in the number of *Nla*IV sites (-a, -b and -c). It therefore seems that the location of DUS relative to homologous regions influences transformation efficacy and that *Nla*IV restriction is not contributing to that particular effect. However, the statistical significance of these differences was repeatedly higher for the transformations in the *Nla*IV background compared to the wt in which the numbers of transformants were considerably lower than for the mutant. This observation suggests that the low transformation frequencies obtained with pDV4-a and pDV1-a due to *Nla*IV restriction partly obscured the DUS location effect, illustrating the importance of delineating individual influences to transformation. This observation suggests that high frequencies of transformation may be used and required to obtain a high enough resolution to delineate subtle effects on transformation. A slightly smaller than two-fold effect from DUS location was considerably more difficult to detect than the effect of restriction which sometimes were larger than fifty-fold, and a number of replicate experiments were therefore undertaken. The significance of this finding remains unknown, but could indicate, in line with another study [29], that more than one level of DUS specificity exist; one at the level of uptake and one further downstream. In any case, further research is required to differentiate between individual processes and their interactions. The influence of DUS location could imply that the DNA region containing the DUS is the region that is first channelled into the cytoplasm and subsequent HR following surface binding. Alternatively, a second level of DUS specificity could be linked to HR in a more direct manner after initial DUS-specific binding and uptake. In any case, the region with DUS position C must provide a higher or more efficient contribution to HR than the DNA region with DUS position A. That contribution is unlikely to be the length of the recombinogenic region since the distance from position C to *ermC* is considerably shorter than the distance from DUS position A to *ermC*. As such, this second level could be the activity of e.g. a DUS-specific nuclease. A third option is that DUS in the C-position facilitates initial surface binding to a better extent than DUS in position A, but how that could come into play is difficult to envisage. Identifying the DUS-specific DNA binding component(s) will lift our understanding of the molecular mechanisms targeting DNA during transformation to a new level and remains a focus for future efforts.

In summary, we have demonstrated how subtle *Nla*IV recognition site deletions, sequence homology manipulations and DUS-alterations exert distinct and opposite effects on meningococcal transformation. These sequence alterations in transforming DNA and RM genes can be used to delineate different stages of and their relative contribution to the transformation process in *N. meningitidis*.

## Materials and Methods

### DNA Constructs

Plasmids and strains employed in the study are listed in Table 2, and primers are listed in Table 3. *Escherichia coli* XL-1 Blue and *N. meningitidis* MC58 were grown as previously described [31]. The hybrid plasmids pOHA-0-DUS and pOHA-Single from a previous study [31] containing the naturally DUS-less neisserial pilus biogenesis gene *pilG* harbouring a selective marker encoding erythromycin (erm) resistance [60], were used to make all constructs employed, varying in DUS, *Nla*IV sites and homologous region as shown in Figure 1. pOHA-0-DUS and pSingle were trimmed with *Bam*HI (recognition sequence GGATCC) (New England Biolabs) and recircularised with T4 Ligase (New England Biolabs) or polished with Phusion polymerase (New England Biolabs) following *Bam*HI restriction and recircularised to generate the plasmids -b, -c and -d versions of pOHA-0-DUS and pOHA13, respectively. These plasmids where used as substrates for PCRs with various combinations of primers 3893OH3_*pilG*3′*Sac*II, 3892OH11_*pilG*5′*Xho*I, 8908OHA3_DUS and 8904OHA11_DUS, listed in Table 3. The resulting PCR products were cloned into the vector pBluescript II SK+ (Stratagene) following *Sac*II and *Xho*I restriction digests of both plasmid and PCR products generating the DUS-variable plasmids (pDV) listed in Table 2. The *Nla*IV knock-out plasmid pOHA1032::*aph* was made by ligating two PCR products with sequence regions within NMB1032 using primers 10324OHA2020, 10325OHA2021, 10326OHA2022 and 10327OHA2023 on either side of the kanamycin resistance gene *aph* (primer 8184OHA_*Aph*EcoRI_REV and 8186OHA_DUS_*Aph*NheI_FOR) and inserting this construct into pBluescript II SK+ using the restriction sites *Xba*I and *Xho*I. Based on pOHA-0-DUS, pOHA-0-DUS-b and pOHA-0-DUS-c three generations of pDV4 and pDV1 that differed in their number of *Nla*IV restriction sites, -a, -b and –c, respectively, were made. pDV4-a and pDV1-a contain the 137 nt *Bam*HI fragment harbouring three *Nla*IV sites at the 3′ end of the *ermC* gene, as shown in Figure 1. In pDV4-b and pDV1-b, 133 nt of the *Bam*HI fragment was removed and the *Bam*HI and a single *Nla*IV site retained, in pDV4-c and pDV1-c this *Bam*HI/*Nla*IV restriction site is also removed. pDV1-c-d1 with a 74 nt deletion near the middle of the left flanking homologous DNA region of pDV1-c, were made using primers 13573OHA2220 and 13574OHA2221 in combinations with appropriate primers, prior to ligation and cloning into vector as before. pDV1-c-d2 and pDV1-c-d3 were made by digesting pDV1-c with *Xho*I/*Bst*XI and *Xho*I/*Psy*I, respectively, prior to generating blunt ends with Phusion polymerase, re-ligation and electroporation into *E. coli* as before. A single C-T transition in the gene encoding the DNA-directed RNA polymerase beta subunit, *rpoB,* causes a His_552_Tyr change and rifampicin resistance in meningococci [61]. A 723 nt DNA fragment capturing this mutation was amplified by PCR using primers OHA2217 and OHA2218, both containing DUS, on a rifampicin-resistant meningococcal DNA template. Intact and *Nla*IV (New England Biolabs) restricted *rpoB* PCR fragments were tested for their transforming abilities. The DNA sequences of all constructs and DNAs were confirmed by DNA sequencing analysis (ABI3130) using primers as shown in Table 3.

### Quantitative Transformation of *N. meningitidis*

The quantitative transformation assays were performed essentially as previously described with 500 ng or 5 ng plasmid transforming DNA or 2.5 ng PCR product, in a bacterial suspension of 0.5 ml consisting of 5% CO_2_-saturated GC broth w/IsoVitalex (typically ten 16 h colonies in 10 ml GC broth to a density of 10^8^ CFU/ml) supplemented with 7 mM MgCl_2_ and incubated for 30 min in 5% CO_2_-saturated GC broth at 37°C [31]. Following the incubations, the bacterial samples were diluted 10-fold in GC broth and grown for 4.5 h before appropriate dilutions were plated onto blood agar medium with or without 8 µg ml^−1^ erythromycin. Transformation frequencies were determined by dividing the number of erythromycin-resistant CFU by the total CFU. Transformation frequencies as low as 8.5 E^−10^ were obtained by plating resuspended pellets from volumes of 4900 µl (1,4 E^11^ total CFU) on single plates with selective medium. Each experiment was repeated at least three times. Two tailed student’s t-tests were used for statistical analyses of the relative differences in transformation frequencies.
